# Reframing adolescent self-harm as a functional continuum

**DOI:** 10.3389/fpsyt.2025.1724449

**Published:** 2025-12-02

**Authors:** Qian-Nan Ruan, Wen-Jing Yan

**Affiliations:** 1 Wenzhou Seventh People’s Hospital, Wenzhou, China; 2 Wenzhou Medical University, Wenzhou, China

**Keywords:** adolescent, functional continuum model, non-suicidal self-injury (NSSI), suicidal behavior, emotion regulation

## Abstract

The clinical and research fields have long relied on a dichotomous classification of adolescent self-harm, rigidly separating non-suicidal self-injury (NSSI) from suicide attempts (SA) based on intent. While operationally convenient for acute risk assessment, this framework oversimplifies the complex clinical reality of mixed and fluctuating intent and obscures the profound developmental and functional links between these behaviors. This perspective article argues for a conceptual shift away from this dichotomy toward a functional continuum model (FCM). The FCM posits that all self-harm behaviors exist on a multidimensional spectrum and are selected from a shared pool of psychological functions—primarily to regulate overwhelming emotional pain (psychache). We reinterpret epidemiological, psychological, and neurobiological evidence to demonstrate that NSSI and SA are not disparate phenomena but rather evolving expressions of the same underlying pathological process. Specifically, NSSI can act as a behavioral training ground that builds the acquired capability for suicide. This model reframes clinical intervention, shifting the focus from mere behavioral cessation to functional rehabilitation—equipping adolescents with adaptive skills to replace the maladaptive functions of self-harm. Finally, we outline future directions for research, including the use of ecological momentary assessment (EMA) and the development of function-specific assessment tools to translate this model into a more precise, dynamic, and compassionate standard of care.

## 1 Introduction

In clinical psychiatry and public health, the classification of adolescent self-harm has been dominated by an intent-based dichotomy that separates non-suicidal self-injury (NSSI) from suicide attempts (SA). This division is not only rooted in assessing intent but also in the historical view of NSSI as a symptomatic expression of other conditions; for instance, it has often been framed as a secondary feature within the diagnostic context of major depressive disorder (1) and viewed as a critical marker of broader developmental psychopathology (2). While this comorbidity-focused view acknowledged NSSI’s complexity, it often relegated the behavior to a secondary feature of a primary diagnosis. The current framework, solidified by systems like the DSM-5, has largely centered on intent, which offers clear operational utility in acute settings and epidemiological tracking (3). When an adolescent presents in an emergency department, assessing suicidal intent is the gold standard for determining immediate safety measures, such as involuntary hospitalization (4). This binary decision point provides a convenient metric for medical records and public health statistics.

However, this operational convenience comes at a significant cost, sacrificing a nuanced understanding of the complex clinical reality. The overreliance on intent focuses attention on the outcome of the behavior rather than its underlying psychological drivers. This not only simplifies the heterogeneity of self-harm but also risks underestimating the danger for high-risk individuals. Attempts to refine this system, such as the proposed suicidal behavior disorder (SBD) diagnosis, still operate within the same categorical framework rather than fundamentally rethinking it (5).

The clinical reality is that adolescent suicidal intent is often ambiguous, fluctuating, and difficult to articulate (6). Many self-harm acts are impulsive, with planning times of less than an hour, occurring during states of intense emotional arousal where the line between wanting to end pain and wanting to end life becomes blurred (7). Our model posits that impulsivity acts as a critical accelerator in the functional escalation from NSSI to SA. When an adolescent’s established coping strategy—NSSI—fails to regulate overwhelming emotional pain, this functional failure can trigger a catastrophic surge in distress and affective arousal. This heightened state of dysregulation overwhelms cognitive control, creating a fertile ground for an impulsive leap to a suicide attempt, which is perceived in the moment as the only remaining option to end the pain. Furthermore, evidence shows that NSSI is sometimes used as a maladaptive coping strategy to prevent a suicide attempt by regulating suicidal ideation (8). This “anti-suicide” function highlights a deep overlap in underlying distress, even if the behavioral goals seem contradictory. The high temporal co-occurrence of NSSI and suicidal thoughts further challenges a clean separation (9). This suggests that a functional escalation occurs when NSSI, an established mechanism for affect regulation (10), fails to contain escalating psychological pain, leading the individual to turn to a more definitive solution—SA.

This progression is strongly supported by epidemiological data. A history of NSSI is one of the most powerful predictors of future suicide attempts, surpassing traditional risk factors like depression or hopelessness (11). The “gateway hypothesis” posits that NSSI actively paves the way for suicide (12). By serving as a “painful and provocative event,” repeated NSSI habituates an individual to pain and fear, thereby building the acquired capability for suicide (13). Thus, NSSI is not merely a correlate but an active, dynamic process that facilitates a lethal escalation.

To address the shortcomings of the current dichotomous model, we propose a functional continuum model (FCM). This model does not seek to discard the role of intent in acute crisis assessment but offers a complementary, integrative framework. The FCM’s core tenet is that all self-harm behaviors, from low-lethality NSSI to high-lethality SA, exist on a continuum, chosen not for their lethality but for the psychological function they are meant to serve. By shifting the primary question from “What is the intent?” (a classification) to “What is the function?” (a process), the FCM provides a more profound, recovery-oriented perspective to guide long-term treatment and dynamic risk prediction.

## 2 Core tenets of the functional continuum model

The centerpiece of the FCM is the assertion that all self-harm behaviors are drawn from a common “functional pool” to manage unbearable internal pain. Validated assessment tools like the Inventory of Statements About Self-injury (ISAS) and the Functional Assessment of Self-Mutilation (FASM) have consistently identified a core set of functions, which are broadly categorized into automatic (intrapersonal) and social (interpersonal) domains (14, 15).

Automatic, or intrapersonal, functions involve the management of internal states and are the most frequently endorsed reasons for self-harm (16). Chief among these is affect regulation, which serves as the central and most prevalent function. Through this mechanism, self-harm acts as a powerful form of negative reinforcement used to “stop bad feelings,” alleviate overwhelming tension, or “calm oneself down” by using physical pain to distract from or neutralize emotional pain (17). Other critical intrapersonal functions include anti-dissociation or grounding, where, particularly for individuals with trauma histories, the pain from self-injury serves to anchor them in reality and combat feelings of numbness. Furthermore, self-harm can serve a self-punishment function, driven by intense feelings of guilt, shame, or self-loathing, where individuals harm themselves to atone for perceived personal or moral transgressions (18).

In addition to managing internal states, self-harm can also serve social, or interpersonal, functions, which involve using the behavior as a means of communication or to influence others—for example, the function of interpersonal communication or influence allows the behavior to “show others how much I am hurting” or to elicit a caregiving response when verbal expression feels impossible or inadequate (19). A different social function involves establishing boundaries and autonomy, where self-harm is used to create psychological distance from others or to assert independence and a rejection of help.

The FCM proposes a critical link between these functions and behavioral escalation. The most lethal suicide attempts are hypothesized to occur when the automatic functions of NSSI—particularly its capacity for affect regulation—catastrophically fail (20). When psychological pain (psychache) intensifies to an absolute and unbearable level and the established coping mechanism (NSSI) no longer provides relief, the individual seeks a permanent escape. In this view, SA is not an unrelated behavior but rather a desperate choice following a profound functional failure. **Table 1** summarizes this shared functional pool.

**Table 1:** The shared functional pool of adolescent self-harm behaviors.

Functional domain	Core purpose/goal	Manifestation in NSSI	Relevance/escalation to SA
Intrapersonal: affect regulation	To reduce/alleviate overwhelming negative emotional states	Cutting, burning, or hitting oneself to generate an immediate emotional shift or relief	Escalation pathway: NSSI fails to regulate extreme pain, leading to a shift toward the “ultimate escape” from feeling
Intrapersonal: anti-dissociation/grounding	To confirm reality or stop feeling numb through physical pain	Deep scratching, pinching, or hitting to counter dissociative and numb states	Implies severe underlying distress (e.g., trauma), which is a risk factor for severe suicidality
Intrapersonal: self-punishment/shame management	To punish the self, manage guilt, and cope with shame	Interfering with wound healing, minor overdoses	Suicide is framed as the ultimate punishment for, or permanent escape from, intolerable shame
Interpersonal: communication/influence	To express internal pain, seek help, or influence relationships	Superficial cutting in visible areas, often accompanied by emotional outbursts	May lead to overt or “performative” attempts intended to force a strong relational response

## 3 Reinterpreting the evidence through a functional lens

The FCM provides a powerful framework for reinterpreting existing evidence from epidemiology, psychology, and neurobiology as a cohesive narrative of a single pathological process. From an epidemiological standpoint, the consistent finding that NSSI is a strong predictor of SA is typically viewed as a risk association (21). The FCM reinterprets this as evidence of behavioral evolution. By serving as a “painful and provocative event,” NSSI provides the behavioral rehearsal necessary for SA. Through repeated self-injury, the individual habituates to pain and lessens their fear of death, thereby building the acquired capability for suicide (ACS) (13). The high epidemiological correlation, therefore, is not a simple overlap of risk factors but a testament to an individual’s progression from a non-lethal coping mechanism to a lethal terminal one.

This behavioral evolution is driven by a unifying psychological driver, psychache, defined by Shneidman as unbearable psychological pain encompassing shame, guilt, loneliness, and fear (22). Within this framework, NSSI is an attempt to manage psychache, for example, by converting it to physical pain, while SA is an attempt to terminate it permanently (23). This functional distinction explains why NSSI can sometimes serve an “anti-suicide” role. Joiner’s Interpersonal Theory of Suicide (IPTS) provides a crucial bridge for this model, positing that suicide results from the convergence of suicidal desire composed of thwarted belongingness and perceived burdensomeness and the ACS (24). The FCM integrates IPTS by proposing that thwarted belongingness and perceived burdensomeness fuel the desire for self-harm, while the specific function shapes its form. Critically, NSSI directly builds the ACS, moving an individual from wanting to die to being able to die. This integration becomes even more powerful when viewed alongside leading stage-based theories like O’Connor’s integrated motivational–volitional (IMV) model of suicidal behavior (25). The IMV model posits a crucial distinction between a motivational phase, where feelings of defeat and entrapment lead to suicidal ideation, and a volitional phase, where an individual moves from ideation to attempt, enabled by factors like acquired capability. The FCM complements and enriches such stage-based models by providing a granular, process-oriented explanation for the transition between these stages.

Within the IMV framework, NSSI plays a unique dual role: its function is to manage the distressing feelings of defeat and entrapment that define the motivational phase, while its practice simultaneously builds the acquired capability for suicide, a key volitional moderator. The critical point of escalation, which we term “functional failure,” occurs when NSSI no longer serves its primary affect-regulation function. This failure catastrophically intensifies the motivational drivers—the sense of entrapment becomes absolute when the only known escape route (NSSI) is blocked. At this moment, the individual is left with unbearable psychache while the acquired capability for suicide is already established. Thus, the functional failure of NSSI acts as the catalyst that propels an individual across the divide from the motivational to the volitional phase, activating the potential for a lethal attempt. Therefore, the FCM adds a unique layer of clinical utility by identifying a dynamic, modifiable process—the efficacy of a person’s coping functions—as a powerful predictor of the imminent transition from wanting to die to trying to die.

This shared psychological process is mirrored by evidence of a shared neurobiological substrate. Neurobiological findings reveal a common vulnerability for both NSSI and SA, primarily involving dysregulation in frontolimbic systems responsible for emotional and behavioral control (26). fMRI studies in adolescents engaging in both behaviors show similar alterations, including reduced volume in the ventrolateral prefrontal cortex and anterior cingulate cortex (27). These regions are vital for top-down emotional control and impulse inhibition, providing a biological basis for the emotion dysregulation that is a hallmark of both NSSI and suicidal behavior. Furthermore, altered pain and reward processing in individuals with NSSI may explain its “addictive” quality as an immediate, negatively reinforcing behavior. These convergent findings support the FCM’s premise that both behaviors arise from a common set of neurofunctional deficits.

## 4 Discussion

Adopting the functional continuum model has profound implications for clinical practice, risk assessment, and future research, mandating a paradigm shift from behavioral management to functional rehabilitation. **Table 2** provides a comparison of these assessment paradigms. This approach demands that long-term treatment planning move from a behavior-centric to a function-centric orientation. The key clinical questions thus become “What psychological function does this behavior serve?” and “What adaptive skills does this adolescent lack to fulfill that function?” The goal is not simply to stop the behavior but to help the individual build an adaptive “functional toolbox” containing functionally equivalent skills such as mindfulness, distress tolerance, and effective help-seeking.

**Table 2:** Contrasting assessment paradigms: dichotomous vs. functional continuum.

Assessment dimension	Traditional intent-focused model	Functional continuum model (FCM)
Primary classification	Intent to die (binary: present/absent)	Psychological function served (spectrum: affect regulation, punishment, communication)
Focus on lethality	Potential lethality of the method (e.g., pill dosage)	Acquired capability (habituation to pain/fear) and intensity of underlying psychache
Risk factors	Static (e.g., past attempts, demographics)	Dynamic (e.g., real-time emotion dysregulation, skill deficits, current interpersonal conflict)
Long-term goal	Behavioral cessation (stopping NSSI/SA)	Functional rehabilitation (building an adaptive skill toolbox)
Acute utility	High (determines immediate need for hospitalization)	Complementary (provides context to predict when functional triggers will escalate risk)

Function-oriented interventions like Dialectical Behavior Therapy (DBT) have already validated this approach, demonstrating significant efficacy in reducing both NSSI and SA by systematically targeting deficits in emotion regulation, distress tolerance, and interpersonal effectiveness (28). This model also complements, rather than replaces, acute risk assessment. By understanding an individual’s primary self-harm functions, clinicians can better predict dynamic triggers for risk escalation, such as when a teen whose primary function is “anti-dissociation” is at highest risk during traumatic flashbacks (29). This shifts assessment from static risk factors to modifiable, dynamic risk processes. Finally, using functional language destigmatizes the behavior by reframing it as a desperate attempt to cope with immense pain rather than a character flaw. This validates the adolescent’s suffering and strengthens the therapeutic alliance.

To translate the FCM from theory into actionable clinical tools, a clear research agenda is needed. First, we must move beyond retrospective reports by employing methodological innovations like Ecological Momentary Assessment (EMA). Using smartphones and wearable sensors, EMA can track the real-time interplay between emotions, situational triggers, and functional urges, allowing us to model the dynamic process of functional escalation with greater precision (30). Second, we must develop and validate function-specific assessment tools. While instruments like the ISAS and FASM are valuable, a streamlined, clinically oriented tool, such as a dedicated Functional Assessment of Self-Harm (FASH) scale, is needed for efficient use in busy clinical settings, ensuring it is culturally adapted and validated for global utility (31). Finally, longitudinal research must investigate the trajectory and stability of self-harm functions over time (32). We need to understand how functions evolve and, crucially, test the FCM’s hypothesis that escalation to SA is preceded by the failure of NSSI’s primary regulatory function.

## 5 Conclusion

The Functional Continuum Model offers a critical evolution in our conceptualization of adolescent self-harm. By integrating evidence from neurobiology, psychology, and treatment science, it provides a more accurate, cohesive, and compassionate framework than the prevailing intent-based dichotomy. It reframes NSSI not as a separate, lesser phenomenon, but as a critical part of a developmental pathway toward acquired capability for suicide. By shifting our focus from “managing behavior” to “healing pain through functional rehabilitation,” we can move toward a more scientifically grounded and deeply humane standard of care, offering more effective support for the mental health of our youth.

## Data Availability

The original contributions presented in the study are included in the article/supplementary material. Further inquiries can be directed to the corresponding author.
